# Cytotoxicity *in vitro* assay in 3D vs. 2D L929 cell cultures – comparative analysis of the response to the latex extracts

**DOI:** 10.1371/journal.pone.0347488

**Published:** 2026-04-28

**Authors:** Beata Gruber-Bzura, Irena Bubko, Lidia Mielczarek, Anna Pogorzelska, Katarzyna Wiktorska

**Affiliations:** 1 National Medicines Institute, Warsaw, Poland; 2 Warsaw University of Life Sciences - SGGW, Warsaw, Poland; Institute of Medical Sciences, Banaras Hindu University, INDIA

## Abstract

The ISO 10993–5 recommends the cytotoxicity *in vitro* tests of the medical devices for the 2D cultures. The aim of the study was: – to develop the L929 cell cultures in the 3D model; – to adapt the above tests for the 3D L929 cell culture using the latex extracts; – to define how the model of the cell culture affect the response of the cells in terms of morphology, viability and the expression of *Bax, Bcl2, Jkamp, Pidd1 and Cyp3a44* genes, and – to check whether this potential response depends on the cell age. To achieve these goals the optical and confocal microscopy, the LDH test and RT-qPCR technique were used. The L929 cells are relatively easy to culture in the 3D model The 10^3–^10^5^ cells/well were selected as optimal for the L929 spheroid formation. Generally, the 3D L929 cell cultures were less sensitive to the latex extracts than the 2D. The visible cytotoxic effects were noted regardless the age of cells, after 48 hrs. However, the spheroids of the older cells, after 72 hrs of exposure neutralized the cytotoxic effects and were regenerated. This effect was also confirmed in the LDH test and may be related to the high expression of the *Bax* gene.The expression of all analysed genes were changed in the 3D conditions. The cytotoxicity *in vitro* studies using the 3D model imposed certain limitations that are not present in the traditional model. Firstly, a limitation is the cell age, as the older L929 cells have been shown to be more sensitive to the cytotoxic agent. Secondly, for the older cells, a limitation may be the exposure time, given the potential for the spheroid regrowth.

## Introduction

The medicinal sciences, and especially the pharmaceutical industry are one of the most relevant fields requiring breakthrough 3D technologies to fill possible gaps between *in silico* hypotheses/*in vitro* results and the *in vivo* settings.The introduction of the new medical devices to the market requires thorough testing, i.e., preclinical testing *in vitro* on a 2D model, *in vivo* animal testing and subsequent stages of clinical testing. This is regulated by the European law, the US, and the internal national regulations, e.g., in Poland it is regulated by the Ordinance of the Minister of Health dated 17 February 2016 [1]. Among other tests *in vitro*, cytotoxicity is the only one that should be conducted for each medical device, independently on the nature of body contact and contact duration. The European Standard ISO 10993−5 [2] is dedicated to the cytotoxicity *in vitro* tests. One of the methods recommended by that document to this purpose (apart from the other two, i.e., direct contact and agar diffusion) is elution test that is done on the extracts of a tested material put on the cell culture. This is the only test dedicated to the most critical materials, i.e., those that are intended for prolonged (>24 hrs to 30 days) or long-term (>30 days) use, for example to the biomaterials (any synthetic ones that are used to replace or restore function to a body tissue and is continuously or intermittently in contact with body fluids) [1]. The *in vitro* tests on a 2D model of cultures do not reproduce the physiological conditions of tissues, which are mostly three-dimensional, which directly affects the biochemistry and biophysics of the system. In turn, animal testing is long-term, very expensive, and constantly raises ethical concerns. For this reason, new research models are constantly sought, which, among others, would provide conditions as close as possible to the physiological ones, while maintaining stability, repeatability and reproducibility of the obtained results [3]. As far as the elution test, due to its specificity, it is the only method mentioned in the ISO 10993−5: 2009 that could be adapted to a 3D cellular model. In the area of the preclinical studies of the medical devices, progress in the development of 3D models mainly concerns the dental products or the products supporting the regeneration of the skeletal system [4–6].The aim of this study was: – to develop and stabilize the L929 cell cultures in the 3D model using agarose, 1.5% as a matrix; – to adapt the elution test for *in vitro* cytotoxicity testing according to the ISO 10993–5:2009 [2] to the 3D L929 cell culture; – to define how the model of the cell culture affect the response of the cells exposed to the latex extracts in terms of morphology, viability and expression of *Bax* (BCL2-associated protein)*, Bcl2* (B cell leukemia/lymphoma 2*), Jkamp* (JNK1/MAPK8-associated membrane protein)*, Pidd1* (p53-induced death domain protein 1)and *Cyp3a44* (cytochrome P450, family 3, subfamily a, polypeptide 44) genes (all of them are related to the cellular response to stress and participate in apoptotic signaling pathways or, like *Cyp3a44,* in xenobiotic metabolism), and, – to check whether the response to the cytotoxic agent depends on the age of the cells. Because the NCTC clone 929 cell line is recommended by the ISO 10993–5:2009 standard [2] and it is used in our routine (accredited) tests, the studies described in this paper were conducted using only this cell line. This line was also chosen due to our empirical knowledge of its reactivity level in 2D.The paper identifies the critical points of the research procedure described in the ISO 10993–5:2009 [2], which, in addition to the culture model, distinguish the conditions recommended for the 3D model, i.e., the preincubation time (for the spheroid formation), the optimal exposure time for the cells to the material (not less than 48 hrs), and the need to use the higher extract concentrations than indicated in the aforementioned standard. A difference in the response to the cytotoxic material (latex), depending on the cell age in the 3D model was also demonstrated.

## Results

### Spheroid formation

The L929 spheroid cultures were produced acc. to Abuelba et al. [**7**] on 1.5% agarose coated the 24- or 96-well plates. The 48-hrs spheroids formed of 10^3^–10^5^ cells/well were selected as optimal to start the elution test in the 3D L929 cell culture. The spheroids made of less than 10^3^ cells/well or formed shorter than 48 hrs were reported as irregular or too small to be easily manipulated during the procedure.The equilibrated spheroid cultures after 48 hrs, at an initial cell density of 10^3^ was confirmed by the relatively low CV diameter of the spheroids formed and the size obtained as optimal for our assays, i.e.,: 300 µm ± 0.1% (CV).

### Elution test acc. to EN-ISO 10993−5 in 2D vs. 3D L929 cell cultures

#### 2D cell cultures.

**Fig 1:**
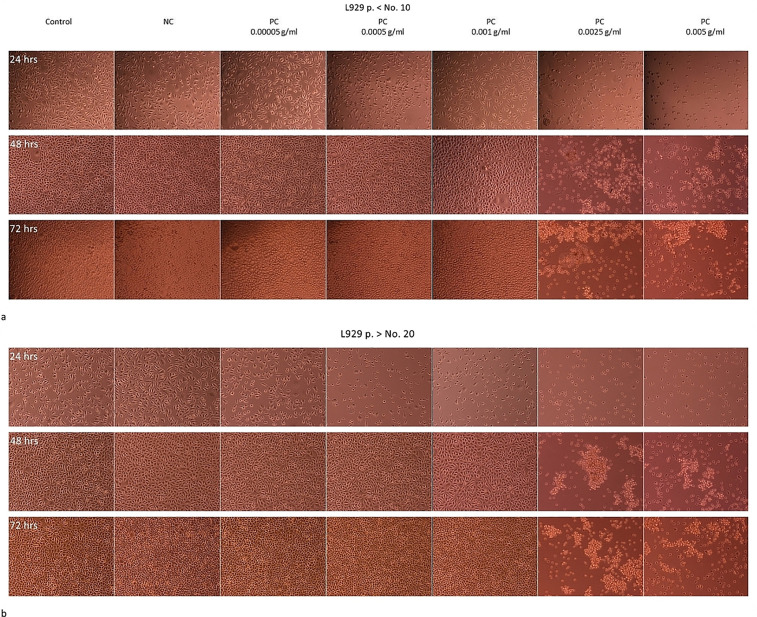
Elution test in the 2D L929 culture. Results of the elution test conducted acc. to the ISO 10993-5 with the L929 cells in the 2D model of culture on the 24-well plates. The old L929 cells, above the 20th passage (**a**) or the young L929 cells, below 10th passage (**b**) were seeded at the density of 5x 10 ^4^ cells/well) and after the 24-hour growth exposed to the extracts of the negative control (NC) or to the extracts of the positive control (PC) at the concentrations from: 0.000025 g/mL to 0.0025 g/mL for 24, 48, or 72 hrs. The images were acquainted with use of the fluorescence microscope Eclipse TS 100F (Nikon) with digital camera Nikon DS-5M-L1, magn. 10x at each time point tested. The results were obtained from n = 2 −3 independent experiments, each done in 4 repeats.

The L929 monolayer culture were exposed to the latex extracts prepared at the concentrations within the range of 0.00005 g/mL - 0.005 g/mL, for 24; 48, or 72 hrs. The concentration range was selected on the basis of the results presented in **Fig 1**, which showed that the 24-hour exposure of the 2D L929 cell cultures to the 0.0025 g/mL was enough to complete destroy the L929 cells.

**Fig 2:**
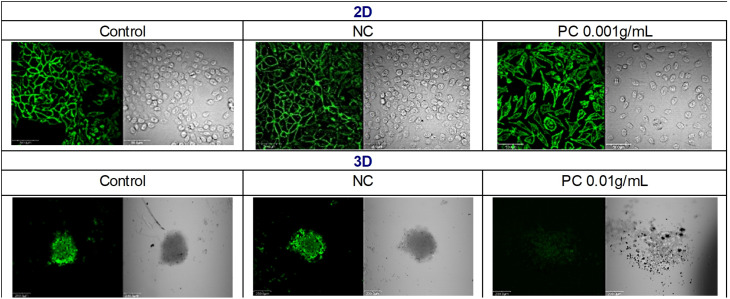
F-actin distribution. The images of the Phalloidin labelled F-actin in the L929 cells cultured in the 2D or 3D model for 24 or 48 hrs, respectively and then exposed for 48 hrs to the negative control (NC) or the positive control (PC) at the concentrations 0.001g/mL for the 2D or 0.01g/mL for the 3D cultures. The untreated cells were treated as the control. The images were acquainted with the confocal microscope Olympus Fluoview FV500. The fixed cells were stained with the Phalloidin-iFluor 488 Reagent for F-actin (green). For the 2D model the scale bars indicate 500 µm and for the 3D model the scale bars indicate 200 µm.

The elution test performed in the 2D L929 cultures demonstrated the appropriate test system response (test correctness), i.e., no cytotoxic response to the extract from the negative control (NC/rubber selected as the working standard) and the expected dose dependent cytotoxic response to the positive control (PC/latex extract), acc. to [**2**]. In the light microscope, the visible inhibition of the culture growth and the drastic change in the cell morphology (shrinkage, massive detachment of the cells from the surface, the loss of shape) were noticeable at the latex extract concentration of 0.0025 g/mL after 24 hrs, regardless of the age of cells. The changes invisible in the light microscope, but visible in the confocal microscope, were noted already at a lower latex concentration, i.e., 0.001 g/mL after staining of the cells with the Phalloidin-iFluor. The Phalloidin binds specifically to the F-actin, enabling its visualization as a green fluorescence. The intracellular distribution of the F-actin in the intact cells is uniform throughout the cytoplasm. In the stressed cells, the protein accumulates in the irregular clusters [8,9]. In this study, in the treated L929 cells we observed the slight intracellular relocation of the F-actin and formation of the irregular F-actin aggregates, particularly visible around the nucleus and along the cell membranes (**Fig 2**).

#### 3D cell cultures.

**Fig 3:**
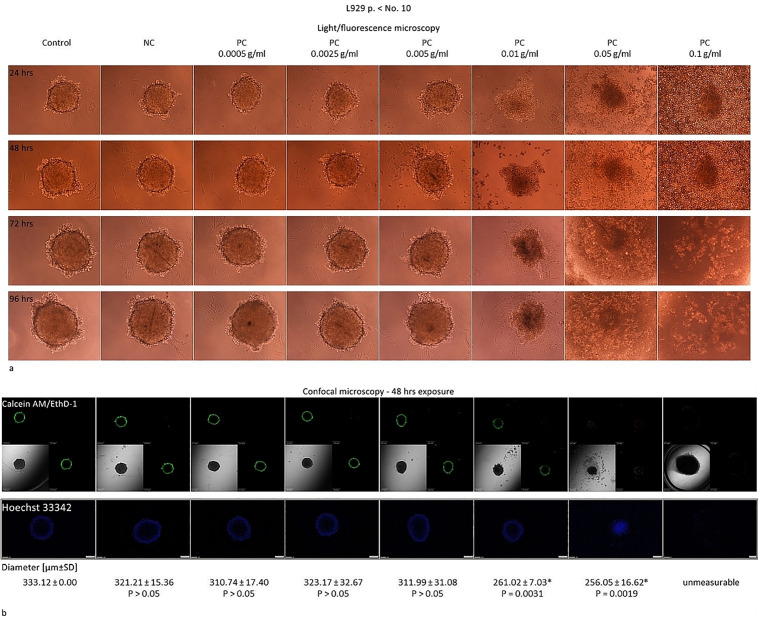
Elution test in the 3D – spheroids from the young L929 cells. Results of the elution test done with the L929 cells in the 3D model on the 96-well plates. The L929 cells were seeded at the density of 10 ^3^ cells/well and after 48 hrs exposed to the extracts of the negative control (NC) or to the extracts of the positive control (PC) at the concentrations from 0.0005 g/mL to 0.1 g/mL for 24, 48, 72, or 96 hrs. The images were acquainted with use of the fluorescence microscope Eclipse TS 100F (Nikon) with digital camera Nikon DS-5M-L1, magn. 10x at each time point tested or with the confocal microscope Olympus Fluoview FV500 with automatic measurement of the diameter of the spheroids [µm ± SD] at the widest section, magn. 20x. *The results with P < 0.05 calculated with t-Student’s test considered as statistically different as referenced to the results obtained for the NC. After incubation at the defined time, the spheroids were stained with the Calcein AM, 2µM, the EthD-III, 4µM (The Viability/Cytotoxicity Assay Kit for Animal Live & Dead Cells) and with the Hoechst 33342, 15µM. As a result, the live cells were stained for cytoplasm (green), nucleus (blue) and dead cells (red). The images from the confocal microscopy show the spheroids and the nuclei at their widest cross-section. The results were obtained from n = 4 independent experiments, each done in minimum 4 repeats.

**Fig 4:**
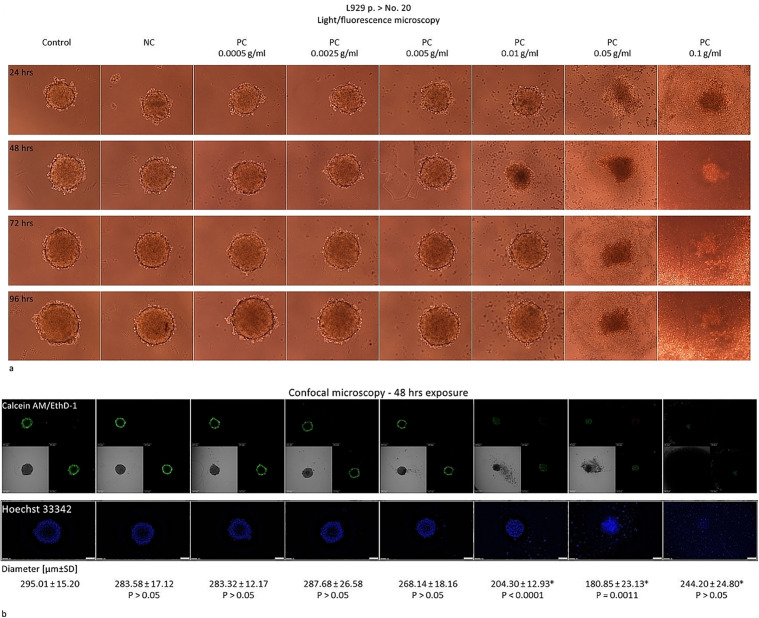
Elution test in the 3D – spheroids from the old L929 cells. The results of the elution test done with the L929 cells in the 3D model of culture on the 96-well plates. The L929 cells were seeded at the density of 10 ^3^ cells/well and after 48 hrs exposed to the extracts of the negative control (NC) or to the extracts of the positive control (PC) at the concentrations from 0.0005 g/mL to 0.1 g/mL for 24, 48, 72, or 96 hrs. The images were acquainted with use of the fluorescence microscope Eclipse TS 100F (Nikon) with digital camera Nikon DS-5M-L1, magn. 10x at each time point tested or for with the confocal microscope Olympus Fluoview FV500 with automatic measurement of the diameter of the spheroids [µm ± SD] at the widest section, magn. 20x. *Results of the exposure to PC with P < 0.05 calculated with t-Student’s test were considered as statistically different as referenced to the results obtained for the NC. After incubation at the defined time, the spheroids were stained with the Calcein AM, 2µM, the EthD-III, 4µM (The Viability/Cytotoxicity Assay Kit for Animal Live&Dead Cells) and with the Hoechst 33342, 15µM. As a result, the live cells were stained for the cytoplasm (green), the nucleus (blue) and the dead cells (red). The images from the confocal microscopy show the spheroids and the nuclei at their widest cross-section. The results were obtained from n = 4 independent experiments, each done in minimum 4 repeats.

To adapt the experimental conditions for the elution test in 3D L929 culture, we also wanted to confirm the test correctness, as was described in the 2D model. The exposure time to the NC or to the PC extracts was in this case as follows: 24; 48; 72 or 96 hrs. The PC extracts were used at the concentration range from 0.0005 g/mL to 0.1 g/mL – the higher than these defined for the 2D model because of the assumed lowered sensitivity of the spheroids [**10**]. The response of the L929 spheroids to the NC or PC extracts was as expected, i.e., the NC extract left the spheroids intact (**Figs 3** and **4**). The PC extracts generated the visible changes in the spheroids starting from the concentration of 0.01 g/mL or the higher, after at least 48 hrs (**Figs 3** and **4**). The clearly visible cytotoxic effects were noted regardless the age of cells and included the reduction of the spheroid diameter and relaxing of the spheroid structure. As a result, the spheroids lost their spherical shape and became irregular. Some cells detached from the main body which was observable as the ragged surface of the spheroids. Interestingly, the spheroids of the old cells, after at least 72 hrs incubation with the PC extracts, 0.01 g/mL neutralized the cytotoxic effects generated after 48 hrs and were rebuilt close to their original dimensions, showing only a slight effect on the surface visible as the single cells detaching (**Fig 4**). On the contrary, in the case of the spheroids of the young cells, the cytotoxic effects generated after 48 hrs of exposure to the PC extracts at 0.01 g/mL or the higher, intensified over time (**Fig 3**). These effects were also confirmed in the LDH test, as described below.

Due to the Phalloidin-iFluor 488 Reagent and the Viability/Cytotoxicity Assay Kit for Animal & Dead Cells, it was possible to visualize the changes in the F-actin organization and the live or dead cells inside the spheroids. The Calcein AM allows to measure the cell metabolic activity. It is an enzymatic fluorescent dye that can passively enter the cells. As an esterase, present in the metabolically active cells, it cleaves the Calcein AM molecules and the calcein is produced which emits green fluorescence. The ethidium homodimer III-1 (EthD-1) selectively penetrates the cells with damaged outer membranes, and marks the dead or necrotic cells in red. The Hoechst 33342 is a membrane-permeable fluorescent dye that can passively enter all nucleated cells and stain the nucleus to emit blue fluorescence [11,12]. The cytotoxic activity of the latex extracts in the spheroids was reflected as: the intracellular reorganization of F-actin and the lowered Calcein AM-derived green fluorescence, that proves the relaxed spheroid structure [**3**, **13** **–** **15**]. As was noted, in the case of both of the spheroids, i.e., formed of the young and of the old L929 cells, the PC extracts at the concentrations of 0.01 g/mL or the higher increased penetration of the Calcein AM into the spheroid (visible as green fluorescence across the widest section) due to the relaxed spheroid structure and the reduced spheroid diameter. As was shown, the outer layer of the spheroids stained red with the EthD-1 what means the dead cells. The analysis of the Hoechst 33342 distribution also showed the relaxed internal spheroid structure – after 48-hour exposure to 0.01 g/mL or 0.05 g/mL, the Hoechst 33342 easily penetrated to the core of the spheroids of the young as well as of the old cells, respectively which was visible as a blue color in the cross-section of the spherical forms (**Figs 3** and **4**). At the PC concentrations below 0.01 g/mL, due to the intact tight intercellular connections, the Calcein AM reached only to the cells placed in the outer layers of the spheroids. The cores remained uncoloured and the size of the spheroids also remained unchanged (**Figs 3** and **4**). The F-actin reorganization could only be assessed in the cells growing as a monolayer (**Fig. 2**). The spheric form made it difficult to observe the intracellular distribution of the actin fibers within the individual cells.

#### Cell viability.

**Fig 5:**
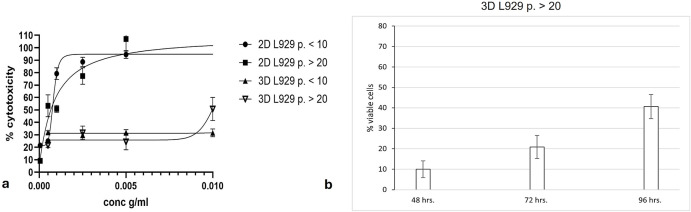
Cell viability. Comparison of cytotoxicity of the latex extracts used at the concentrations from 0.00005 to 0.005 g/mL (2D) or from 0.0005 to 0.05 g/mL (3D) in the young or in the old L929 cells. The 24-hour monolayer cultures or the 48-hour newly formed spheroids were exposed to the latex extracts for 48 hrs and after that time subjected to the LDH test (CytoTox 96®Non-Radioactive, Promega Corp.) **(a)**; regeneration of the spheroids from the old cells, exposed to the latex extract, 0.01 g/mL for 48 hrs, 72 hrs or 96 hrs and after that time subjected to the LDH test (CytoTox 96®Non-Radioactive, Promega Corp.) **(b)**. Data are expressed as the mean ± SD of n = 3 independent experiments, P < 0.000001 for all experiments The results were analysed using GraphPad Prism software.

The LDH test is based on the measurement of the lactate dehydrogenase (LDH) activity, an enzyme released during the cell lysis. The test mechanism consisted in the enzymatic conversion, with the participation of LDH, of a tetrazolium salt (INT) into the red formazan, the absorbance of which was measured. The aim of the cell viability study was a comparative analysis of the response of the L929 cells growing in a 2D or 3D model to the PC extracts, depending on the age of the initial culture. Depending on the model, the cells were exposed for 48 hrs to the PC extracts at the concentration range of: 0.00005–0.005 g/mL for 2D or 0.0005–0.05 g/mL for 3D. As was shown, the IC_50_ for the PC/latex, defined for the spheroids of the old L929 cells reached nearly 0.01 g/mL, which is consistent with the morphological assessment, while the spheroids of the young L929 cells were statistically less sensitive (0.00823 ± 0.00147 g/mL *vs*. 0.00879 ± 0.00295 g/mL, P < 0.000001, respectively) (**Table 1**, **Fig 5a**). For the 2D cultures of the old L929 cells, the lower concentrations of the PC extract was sufficient to inhibit 50% of the cell culture than for the young cells (0.00061 ± 0.00009 g/ml *vs*. 0.00069 ± 0.00006 g/ml, respectively, P = 0,403997). So, the spheroids of the young cells showed approx. 12.7 times lower sensitivity compared to the young cells cultured as a monolayer (P < 0.00001) (**Table 1**). In the case of the old cells, the spheroids were approx. 13.5 times less sensitive than the old cells cultured as a monolayer (P < 0.00001) (**Table 1**). Fig 5 **a** shows that the most rapid reaction to the latex was observed in the young cells cultured as a monolayer. A very interesting effect occurred in the spheroids of the old cells after the 72- hour and the 96-hour exposure to the latex extract, 0.01 g/mL. As was presented on **Fig 5 b**, the number of the viable cells started to increase from the level of the complete cell culture destruction up to 21% and 42% of the live cells noted after 72 and 96 hrs, respectively. It was also observable as a spheroid reconstruction (**Fig 4**). Such effect was not noted either in the spheroids made of the young cells or in the monolayer cultures.

**Table 1:** IC50 values (± SD) determined for the PC extract in the NCTC clone 929 cells, depending on the cell age and culture model.

	2D p. < 10	2D p. > 20	3D p. < 10	3Dp. > 20	3D/2D < p.10	3D/2D > p. 20
**IC**_50_^*^ **[g/mL]**	0.00069 ± 0.00006	0.00061± 0.00009	0.00879± 0.00295	0.00823± 0.00147	12.7 P < 0.000001	13.5 P < 0.000001
**R** ^**2**^	0.9062	0.8863	0.7608	0.8665	–	–

*The 24-hour monolayer cultures or the 48-hour newly formed spheroids were exposed to the PC extracts for 48 hrs (2D, 3D), 72 or 96 hrs (3D) and after that time subjected to the LDH test. Data are expressed as the mean ± of n = 3 independent experiments, P < 0.000001 for all experiments.

#### RT qPCR.

In this part of study, the cultures were carried out analogously to the scheme in the elution test. The PC extract concentrations were selected as the last before the concentrations generating the cytotoxic effects, i.e., 0.001 g/mL (for RT-qPCR 0.0005 g/mL) for 2D cultures and 0.01 g/mL (for RT-qPCR 0.005 g/mL) for 3D cultures.

**Fig 6:**
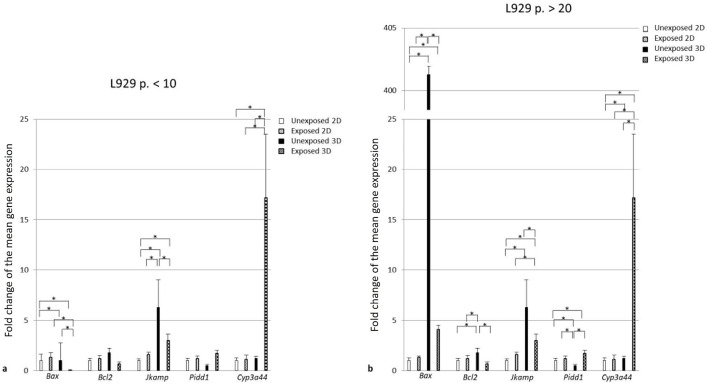
Gene expression. Expression of *Bax*, *Bcl2*, *Jkamp*, *PIDD1*, and *Cyp3a44* genes determined by RT-qPCR in the L929 cells cultured in the 2D or the 3D models for 24 or 48 hrs, respectively and then exposed for 48 hrs to the latex extract, 0.0005 g/mL (2D) or 0.005 g/mL (3D). The cultures established of the young L929 cells **(a)**. The cultures established of the old L929 cells **(b)**. Results are presented as the fold change (FC) of the mean gene expression ± SD, regarding the normalizer gene β-glucuronidase (GUSB). The data are expressed as the mean of n = 2 or 3 independent experiments. * P < 0.05. The results were analyzed using Tukey’s multiple comparison test**.**

In the 2D model, the expression gene profiles did not change significantly under the PC extract, regardless of the cell age. The cell age also did not differentiate the expression of these genes as a constitutive feature (Fig 6a-6b**)**.

In the 3D cultures, the *Bax* gene was characterized by a much higher (above 400-fold) constitutive expression in the spheroids of the old cells than of the young cells (P < 0.0001). As compared to the 2D cultures of the young cells, the spheroids of the young cells showed the two-fold or the five-fold higher constitutive expression of two genes, *Bax* and *Jkamp*, respectively (P = 0.0234 and P < 0.0001). For the spheroids of the old cells as compared to the 2D cultures of the old cells, the lower constitutive expression of *Pidd1* and the higher constitutive expression of the rest of the tested genes were noted (P < 0.0001 for each) (**Fig 6a, 6b**). In the spheroids of both, the young and the old cells exposed for 48 hrs to the latex extract, 0.005 g/mL, the significant changes in the expression of most of the tested genes were observed such as: for the young cells: *Bax* ca. two-fold upregulation, P = 0.0009; *Jkamp*, ca. two-fold deregulation, P < 0.0001 and *Cyp3a44*, ca. 18-fold upregulation, P = 0,0001 and for the old cells: *Bax* ca. 50 – fold deregulation, P < 0.0001, *Bcl*-2 above two-fold deregulation, P < 0.0001; *Jkamp*, ca. two-fold deregulation, P = 0.0002, *Pidd1* above seven- fold upregulation, P < 0.0001 and *Cyp3a44*, ca. 17-fold upregulation, P < 0.0001 (**Fig 6a, 6b**).

## Discussion

In the context of the medical devices, the development of the three-dimensional structures is progressing relatively slow and the number of the available, well-characterized studies on the cell models that could be used in the assessment of cytotoxicity is small. There is a particular lack of adaptation of the standard procedures – such as tests in accordance with the ISO 10993−5 – to the 3D conditions.

The authors of this paper attempted to develop a 3D model of the L929 mouse fibroblasts forming the spheroids in 1.5% agarose and assess the possibility of its use in the elution test as a reliable alternative to the classic 2D test in the assessment of cytotoxicity of the medical devices. According to Baciu et al. [**16**], obtaining the repeatable spheroids at the low cost is crucial for conducting the *in vitro* screening studies. The L929 cell line was tested for the ability to form the 3D structures on different carriers, e.g., in a collagen hydrogel (collagen type I at a concentration of 3.0 mg/mL), which is also a costly process [**17**]. The adaptation of the 3D cell culture conditions with the method described by Abuelba et al. [**7**] on the agarose 1.5% coated plates turned out to be the right to receive the L929 spheroids. The agarose 1.5% occurred the appropriate matrix for this cell line to get the regular spheroids and it is consistent with Ghanta et al. [**10**] who did it with the L929 cells on agarose 1%. The initial cell number/well (10^3–^10^5^) was similar as proposed by these authors and close to that used by Sambale et al. [**14**] for the other murine cell line, NIH-3T3 (2.5x10^3^ cells/well). In our study, it was possible to obtain the regular spheroids during 48 hrs, from as few as 10^3^ cells per well, reaching a diameter of about 300 µm each, i.e., within the optimal range for research on the cell response to the different agents [**14**]. In contrast to the results obtained by Brochado et al. [**18**] on the spheroids formed of MC3T3-E1 (preosteoblastic murine cells), in our study the correlation between the culture time of the spheroids and their diameter was positive while Brochado et al. [**18**] noted an inverse correlation, explaining this phenomenon progressing the compaction process. The matrix used in the cited work was 1% agar. On this base, our results may suggest the cell specific differences in the internal structure of the spheroid and the degree of cell packing. As Sambale et al. [**14**] and Sirenko et al. [**15**] reported, the small-sized spheroids, i.e., from 5 x 10^2^ or less cells were observed to have less consistent spheroidal shape and did not display the complexity of real tissue, which was also noted during our preliminary experiments. Some results noted by other authors revealed the quite different efficacy and rate in the formation of the spheroids by the L929 cells even using the same type of matrix, such as agarose, that is difficult to explain. For example, Bozhok et al. [**19**] reported that, the L929 cells at the initial density 10^5^ cells/mL or 2x10^5^ cells/mL on the agarose 2%, during 3 days formed only the single small spheroids of the diameters: 5–10 µm for 10^5^ cells/mL and 30–40 µm for 2x10^5^ cells/mL. The agarose solution, 2% was also used to obtain the 3D structures from the L929 cells, referred to by Andrade et al. [**20**] as the microtissues (MT). As compared to our studies, these 3D structures formed much slower and a more distinct MT shape was observed from the seventh day. In our study, the L929 cells/mL at a density of 10^4^/mL, during 48 hrs formed the regular spheroids, of diameters 420–460 µm in 1.5% agarose. It may be suggested that the agarose concentration play a role in more or less efficient cellular aggregates formation and that the more agarose concentration, the lower ability of cells to form spheroids. As we’ve noted, the L929 cells formed the spheroids very easy, starting the aggregation as early as after 5 hrs regardless of the cell age. Dynamics of the spheroid formation in the L929 cells was similar to that, observed by Sambale et al. [**14**] in the other mouse fibroblasts cell line, NIH-3T3 which formed the regular spheroids on the 96-well, ultra-low attachement plates after 6 hrs.

The other goal of this study was to transfer the elution test acc. to the ISO 10993−5, routinely made with the 2D cell cultures, to the 3D L929 cell cultures. To obtain the equilibrated spheroid culture, we proposed the initial number of the L929 cells as 10^3^ cells per well and the preincubation time as 48 hrs. We also confirmed that the 24 – hour exposure of the L929 spheroids to the extracts was sufficient to develop the observable cellular response. The confocal microscopy allowed to distinquish the viable and the dead cells more insight that is not possible to observe with the light microscopy. Because of the condensed structure of the spheroids, the potential dead cells do not have to separate from the spheroid body and then no significant visible changes is observed, even under the influence of the cytotoxic agent [**13**]. Above twelve-fold less sensitivity of the L929 spheroids to the latex extract, regardless the age of the L929 cells was consistent with the effect observable by Restle et al. [**3**] on the example of the 2D and the 3D culture of the murine pre-osteoblasts cells (MC3T3-E1) exposed to, among others, the latex. In the current study, the spheroids untreated or exposed to the latex extract in the non-cytotoxic concentrations stained with Calcein AM in the outer layer only. The spheroids after exposure to the latex extracts which induced disruption and relaxation of the spheroid structure, showed green fluorescence across the entire section. Such interpretation is consistent with Frandsen et al. [**21**] who used Calcein to visualize the cytotoxic effects in the human bladder cancer spheroids and the human dermal fibroblast spheroids. According to the authors, unstained areas of the spheroids were the result of difficult penetration of Calcein AM due to the tight intercellular junctions.

As Souza et al. [**22**] suggested, the difference between the results of cytotoxicity assay obtained with the 2D and the 3D cell cultures may result from the different architecture of the cell culture models. The authors propose the several hypotheses to explain the chemoresistance observed in the 3D culture, including, among others, poor diffusion of the compounds in the cellular structure, alteration of the receptor proteins, the drug transporters, and the enzymatic metabolism activity. The drug response in the 3D cell cultures may be also affected by the differences in the cell number and the serum concentration [**23**]. In our studies, the used FBS (fetal bovine serum) concentration (10%) was the same, but the cell number seeded on the plates in the 2D cultures was 50-fold higher than in 3D (5x10^4^ vs^.^10^3^). Nevertheless, according to the authors of this paper, the type of culture is the most important. According to Lee et al. [**13**], densely packed cells in the spheroids are covered by a well-developed layer of ECM (extracellular matrix) that reduces the penetration of the toxicants. Hence, the inner layer of the cells receive less damage than the cells in the outer layer. Also, the dead cells on the outer layer of the spheroid potentially act as a temporal protective barrier against the toxic materials as they increase the thickness of the ECM.

In this study, apart from the reduced sensitivity of the L929 spheroids to the latex extract in comparison with the L929 monolayer, an effect indicating a reversible sensitivity of spheroids from the old cells (above passage 20) to the latex extract concentration of 0.01 g/mL was noted that was also confirmed by a visual observation. Such reconstruction was not observed at the higher extract concentrations, i.e., 0.05 or 0.1 g/mL. We also noted that in the case of the spheroids from the young cells, the cytotoxic effects of the extracts, 0.01 g/mL definitely deepened over time (up to the end of the tested 96 – hour incubation). So, this effect could not be a consequence of neutralization of the cytotoxic compounds in the extract itself. Selectivity of this effect based on a specific concentration and the time after which it was noted resembles the effects observed by Zingales et al. [**24**] in the studies on the sensitivity of the 2D and the 3D cultures of the SH-SY5Y and the MDA-MB-213 cancer cells and the BM-MSC and the HUVEC normal cells to the selected mycotoxins. The authors observed the lack of the linear dependence of the spheroid sensitivity on both, the time of exposure and the concentration level of a given factor.

As described in the publication by Gomes et al. [**25**], after the cytotoxic effects during treatment of the cells in both the 2D and the 3D models with a cytotoxic compound, there may be a return to the proliferative capacity or the permanent damage to the cells, which leads to death. Generally, the possible regeneration of the spheroids may have several reasons, e.g., a well-developed protective ECM layer or formation of an additional protective layer of collagen and proteoglycans (produced, for example by L929 spheroids) [13,17,26–28]. Also the L929 cells secrete M-CSF (macrophage colony-stimulating factor), which can promote the regeneration and the growth of the spheroid core cells, less exposed to cytotoxic factors [**29**]. In our studies, the observed effect as a spheroid regeneration was dependent on the cell age and concerned only the relatively old cells, i.e., being above passage 20 at the cell seeding stage. This, so far, on the basis of this study we are unable to explain. It seems that the L929 probably changes its properties over time with increasing the passage number above 20, and the resistance of spheroids, observed here as the reconstruction of the spherical structure during exposure, is closely related to the concentration of the cytotoxic agent. Based on these observations, it can also be suggested that spheroids composed of the younger cells show a lower ability to adapt to the external conditions and will be more sensitive to the cytotoxic factors. Further research will be required to explain the mechanisms of the observed phenomenon of regeneration of the spheroids formed from the older cells.

The similar constitutive expression of the *Cyp3a44* gene in the L929 2D and 3D cultures, and the significant increase in the expression of this gene in the intact spheroids of hepatocellular carcinoma, HepG2 noted by Liao et al. [**30**], suggest the cell specificity in the cell adaptation to the type of culture. At the same time, it is worth emphasizing the significant increase in the level of *Cyp3a44* expression in the L929 spheroids as compared to the monolayer culture under the influence of the latex extract, regardless the cell age. This effect may be part of the rationale for the lower sensitivity of the spheroids to this agent, because *Cyp3a44* is a gene responsible for phase I metabolic processes involved in the elimination of xenobiotics. Using 2D cultures of liver cells as an example, Luckert et al. [**31**] indicated that the high potential to metabolize xenobiotics is strongly decreased or even lost under the 2D culture conditions. The relatively low expression of *Cyp3a44* obtained in our studies in the 2D L929 cultures, with a simultaneous lack of gene response to the latex extract also points to the lower potency of the 2D cultures in metabolizing of the foreign substances and, the same, it may prove Luckert’s et al. [**31**] observation. According to Fontoura et al. [**32**] for the reduced sensitivity of the 3D cultures, the cell-cell and the cell-matrix interactions are important. He et al. [**33**] in both, the colon cancer cells HCT116 and the LoVo cells treated with platinum, noticed the reduced expression of the p53 gene in 3D cultures. According to the authors, the 3D architecture has an impact on the decreased chemosensitivity to platinum and the *p53* gene is involved in its mechanism. In our studies, we noted the opposite effect observed as the increase of the *Pidd1* gene expression (the functional equivalent of the p53 gene in mice), but only in the old cells which confirms, firstly, the cell dependence of the stress response and, secondly, the dependence of the stress response on the age of the cells. The cell age-dependent reversibility of the stress response, observed in our studies coincided with a significant reduction of the expression of *Bax* gene.

All procedures related to the tests for biocompatibility *in vitro* are conducted in the 2D cell cultures while the cells naturally grow in a 3D environment which is close to the natural conditions. As a result, the tests done on the monolayer cell cultures sometimes provide misleading and the nonpredictive data for *in vivo* responses [**34**]. The interactions in the 3D spatial arrangement impact the cellular functions, providing more physiologically relevant information on the cell growth and as a consequence, determine the cellular responses to the external stimuli [**35–37**].

## Conclusions

The conducted studies show that the L929 cells are relatively easy to culture in the 3D model.The L929 cell cultures, initiated on the 1.5% agarose have the form of the regular spheres that seem to be a good object for research on the influence of the cytotoxic agents on the cells. However, cytotoxicity *in vitro* studies using a three-dimensional model imposed certain limitations that are not present in the traditional model. Firstly, a limitation is the cell age, as the older L929 cells have been shown to be more sensitive to the cytotoxic agent and, secondly, if the older cells are used in the study, the exposure time may be another one limitation, given the potential for the spheroid regrowth. The regrowth did not occurred in the 2D model. Therefore, it can be assumed that the 3D model significantly alters the conditions for the cell growth and adaptation to the external factors, which is likely related to the constitutive expression profile of the specific genes. Given that the 3D culture model creates the conditions for the cells that are much more realistic, it seems more appropriate for assessing the biocompatibility of medical devices *in vitro*. An additional benefit of using the 3D model is the possibility of the long-term toxicity testing that was emphasized by Lee et al. [**13**]. It should be also remembered that in the 3D cultures, the concentrations of the latex extract and the sample itself should be verified, depending on the type of the cells used in the study.

The further studies in this area are fully legitimate. Among other things, it is about the comparative analysis of the profiles of the stress-related genes and proteins dependently of the type of matrix. The results obtained in this work will be the basis for the further studies.

## Materials & methods

### Cells

Cells were purchased from the American Tissue Cell Culture Collection (USA): the NCTC clone 929 (L929) – the mouse areolar fibroblasts from subcutaneous connective tissue (ATCC-CCL-1, USA). The cells were cultured in a humidified atmosphere at 37 °C in 5% CO_2_ in the Minimum Essential Medium (MEM) with L-glutamine (Biowest), supplemented with the 10% heat-inactivated fetal bovine serum (FBS) (Biowest) and the Antibiotic Antimycotic (Biowest) (0.100 units penicillin, 0.1 mg streptomycin and 0.25 μg Amphotericin B per mL). All culture media were pre-warmed to 37 °C before use. The cell cultures used in the studies were mycoplasma free as was routinely tested according to our own protocol with the qPCR technique. For the purpose of this study, the 2D and the 3D L929 cell cultures were initiated from the cells being at the passages below No. 10 (young cells) or above No. 20 (old cells) after defrosting. All passages tested did not exceed No. 30.

### 2D cultures

The L929 cells at a density of 5x10^4^/mL were seeded in the 24-well plates and cultured for 24 hrs in a humidified atmosphere, 37°C and 5% CO_2_ to form a monolayer.

### 3D cultures – spheroid formation

The L929 cells were seeded on the 1.5% agarose coated 96-well plate at the density 10^3^/well, covered with the MEM with the 10% FBS and incubated for 48 hrs to form the regular spheroids. Prior the seeding, the wells were coated with warm 1.5% agarose (Sigma-Aldrich) in the PBS (phosphate buffered saline)(IITD, Wrocław, Poland), 70 µL and left to solidify, for about 20 minutes [**7**].

### Elution test

Regardless on the model of culture, to confirm the correctness of the test, in each elution test the following controls were used: the intact cell culture as the reference culture and the culture exposed to the extract from the NC (the material when tested in accordance with the ISO 10993−5: 2009 does not produce an cytotoxic response). The negative material used in this study was rubber. Due to the fact that the test sample was latex, a material with recognized severe cytotoxicity, the additional PC (the material when tested in accordance with the ISO 10993−5: 2009 produces an cytotoxic response) was not used.

### Extract preparation

The extractant used in this study was the MEM with the 10% FBS. The appropriate aliquots of the NC and the PC were immersed in the extractant and incubated for 24 hrs, at 37°C [**6**]. The latex as the PC was selected because of the recognized cytotoxicity being the good choice of the material to validate the elution test with use of the spheroids.

### Exposure to the extract

2D. The 24-hour monolayer L929 cell cultures were exposed to the 24-hour PC extracts of the selected concentrations for 48 hrs. The prepared extracts were placed in the appropriate wells with the monolayer culture in place of the culture medium for 48 hrs in a humidified atmosphere, at 37°C and 5% CO_2_. After this time, the cultures were assessed for morphology, intracellular distribution of the F-actin and the expression of *Bax, Bcl2, Jkamp, Pidd1 and Cyp3a44* genes. The response of the monolayer cultures exposed to the extract was referenced to the intact control.

3D. The 48-hour L929 spheroids were exposed to the 24-hour PC extracts of the selected concentrations for the next 48 hrs. The prepared extracts were placed in the appropriate wells with the spheroids on the 1.5% agarose layer in place of the culture medium for 48 hrs in a humidified atmosphere, at 37°C and 5% CO_2_. For this purpose, the MEM from the wells was carefully removed so as not to damage the spheroids and replaced with the appropriate extracts. The spheroids were exposed to the extracts for 24; 48 or 96 hrs. After this time, the spheroids were assessed for morphology, intracellular distribution of the F-actin and viability and expression of *Bax, Bcl2, Jkamp, Pidd1* and *Cyp3a44* genes. The response of the spheroids was referenced to the intact control.

### Morphology evaluation and staining procedure

The 2D and 3D cell cultures were evaluated using the light/fluorescence microscope Eclipse TS 100F (Nikon) with digital camera Nikon DS-5M-L1 or the confocal microscope Olympus Fluoview FV500 and the fluorescence microscope Olympus IX70. The confocal microscope made possible to measure the spheroid diameters automatically, at the widest section and to observe the F-actin distribution or the live and the dead cells within the spheroids. For this type of microscope, the cells were cultured on the µ-Plate Angiogenesis 96 Well (ibidi GmbH, Germany).

The events, such as the F-actin distribution or location of the live or the dead cells within the spheroid, were possible to follow after staining with the Phalloidin-iFluor 488 Reagent (Abcam) or a mixture of three dyes, as the Calcein AM (2 µM) and the EthD-1 (4 µM) (Viability/Cytotoxicity Assay Kit for Animal & Dead Cells, Biotium) and the Hoechst 33342 (15 µM) (Sigma-Aldrich). In the first case, to stain the F-actin with a Phalloidin-iFluor, the cells were fixed with 3% methanol-free formaldehyde in the PBS (Phosphate Bufferred Saline, IITD, Wroclaw, Poland) to avoid disruption of an actin, next permeabilized with 0.1% Triton X-100 for 3 minutes and then stained with the 1X Phalloidin-iFluor 488 Reagent for 90 minutes. Mixed dyes solution (Biotium), prepared finally in the PBS was prepared immediately before use and added to the wells, without aspiration of the MEM to avoid damage or removing of the cells or the spheroids. Incubation time for staining was 2.5 hrs. The staining procedure was optimized according to Sirenko et al. [**15**].

### Cell viability

LDH test. For this purpose, the CytoTox96®Non-Radioactive (Promega Corp.) was used. 2D: The cell suspension was seeded at 5x10^3^ cells/ml on the 24-well plates and cultured in the MEM with the 10% FBS for 24 hrs. After this time, the cultures were exposed for 48 hrs to the 24-hour latex extracts at the concentrations within the range: 0.00005–0.005 g/mL, and then the LDH test was performed according to the manufacturer’s instructions. 3D: The cell suspension was seeded at 10^5^ cells/mL in the 24-well plates covered with a layer of 1.5% agarose in PBS, and then cultured for 48 hrs in the MEM with the 10% FBS to form the spheroids. After this time, the newly formed spheroids were exposed for 48 hrs to the PC extracts, at the concentrations within the range: 0.0005–0.05 g/mL. Then, the LDH test was performed according to the manufacturer’s instructions. The absorbance of the red formazan formed from tetrazolium salt by the enzyme released by the lysed cells was measured at a wavelength of 490 nm using a BIOTEK microplate spectrophotometer. The percentage of the dead cells in the analyzed culture was calculated according to the formula: Dead cells (%) = 100% × (Experimental LDH release, %/Maximum LDH release, %), where the maximum LDH release means the result of the test consisting in the analysis of the LDH release from the intact cells, exposed to the lysis buffer. The background absorbance was subtracted from all readings obtained as a result of the measurements. The raw data analysis included the absorbance readings obtained in n = 2–3 independent experiments. Statistical significance was assumed at P values < 0.05.

Statistical analysis was performed using GraphPad Prism 7 software (Graph-Pad Software, San Diego, US).

### RNA isolation and gene expression analysis

#### 2D.

The L929 cells were cultured in the Nunc flasks and seeded at a density of 1.5 x 10^6^ per flask. After the 24 – hour growth the cultures were exposed for 48 hrs to the PC extract at a concentration of 0.0005 g/mL. After this time, the cells were trypsinized, centrifuged at 2000 rpm, washed with PBS and centrifuged again. The resulting pellet was the material for RNA isolation with use of the kit cat. No. E3599, EURx. The RNA isolation was performed according to the manufacturer’s instructions. The isolated RNA was stored at −70 C.

#### 3D.

The L929 cells were seeded at a density of 10^5^cells per well on the 24-well plates and after the 48 – hour growth the formed spheroids were exposed for 48 hrs to the PC extract at a concentration of 0.005 g/mL. After this time, the spheroids were collected, centrifuged at 2000 rpm, washed with PBS and centrifuged again. The resulting pellet was the material for RNA isolation with use of the kit cat. No. E3599, EURx. The RNA isolation was performed according to the manufacturer’s instructions. The isolated RNA was stored at −70 C.

The isolated RNA was stabilized with the RNAse inhibitor (cat. No. E4210-01, EURx) (2µl per 50 µl RNA) and next, purified from the residual DNA according to the manufacturer’s instructions (kit cat. No. K2981 Thermo Scientific). The ingredients were mixed, vortexed and incubated for 30 minutes at 37°C in a thermocycler. For each unit of DNase I, 2 μL of the DRR reagent was added.The mixture was incubated at room temperature for 2 minutes with gentle stirring 2–3 times to resuspend the DRR reagent. Next, the tube was centrifuged at ≥800 g for one minute to pellet the DRR. The supernatant was transferred into a new tube. Next, the RNA was transcribed by reverse transcription into the cDNA during the cDNA synthesis reaction, according to the manufacturer’s instructions (kit no. RR037A, Takara). The ingredients were mixed, vortexed, incubated for 15 minutes at 37°C, next, for 5 seconds at 85°C in a thermocycler and moved to the 4°C (the 500 ng of the purified RNA was reverse transcribed in 10 μl of the reaction mixture). The cDNA was stored at −20 C. The following protocol was used to prepare the reaction: Real-Time PCR using RT2 qPCR PrimerAssays and RT2 SYBR® Green Mastermixes/Handbook (Qiagen). The mouse gene primers selected for analysis were purchased from Qiagen: *Bcl2* (catalog No. 330001; PPM02918F-200), *Jkamp* (catalog No. 330001; PPM37131A-200), *Bax* (catalog No. 330001; PPM02917E-200), *Pidd1* (catalog No. 330001; PPM04964A-200) and *Cyp3a44* (catalog No. 330001; PPM57660E-200) and the normalizer gene primer *Gusb* (β-glucuronidase) (catalog No. 330001;(PPM05490C-200). The reaction was performed using RT2 SYBR Green ROX MasterMix (catalog No. 330521), with the Stratagene Mx3005P apparatus. The experiment was performed according to the RT2 qPCR Primer Assay Handbook. The concentration of cDNA was 5 ng/one reaction/one test tube. The raw data analysis included the Ct values obtained in n = 2–3 independent experiments for the individual genes. Raw data from the Stratagene Mx3005P instrument were analysed as was explained in **S3 File Supporting information.** Tuke’s multiple repetition test was used to statistically analyze the results. Statistical significance was assumed at P values < 0.05.

## Supporting information

S1 Table Cell viability of L929 cells. (PDF)

S2 Fig Normalized mean FC and SEM values. (PDF)

S3 File Supporting Information. (PDF)
