# Supplementary material for: Cytotoxicity in vitro assay in 3D vs. 2D L929 cell cultures – comparative analysis of the response to the latex extracts
Source: PLoS One. 2026 Apr 28;21(4):e0347488. doi: 10.1371/journal.pone.0347488 (PMC13123997; doi:10.1371/journal.pone.0347488)
Supplement: S1 Table — (PDF) [file pone.0347488.s001.pdf]

S1 Table. Cell viability of L929 cells cultured in 2D and 3D systems after exposure to different concentrations of the tested compound (**a**). The normalized data that were used to calculate the mean values presented in Table above (**b**).

**log(conc)**	**2D culture**		**3D culture**	
	L929 p < 10 (Mean ± SD)	L929 p > 20 (Mean ± SD)	L929 p < 10 (Mean ± SD)	L929 p > 20 (Mean ± SD)
-1.00	100.00 ± 0.92	100.00 ± 1.05	100.00 ± 2.44	100.00 ± 4.16
-1.30	96.00 ± 3.39	107.02 ± 9.69	100.88 ± 2.46	100.00 ± 1.23
-2.00	—	—	31.62 ± 3.17	50.73 ± 9.27
-2.30	94.54 ± 3.13	107.02 ± 9.69	31.86 ± 2.28	24.31 ± 6.39
-2.60	88.71 ± 3.65	77.29 ± 6.63	29.76 ± 2.26	31.61 ± 5.44
-3.00	79.26 ± 4.68	50.80 ± 2.77	—	—
-3.30	24.97 ± 0.16	53.31 ± 8.82	31.93 ± 1.30	21.49 ± 1.71
-4.30	21.53 ± 0.47	9.08 ± 0.46	—	—

a

Log (conc)	2D p<10						2D p>20						3D p<10						3D p>20					
-1.00	98,73	99,37	99,68	100,32	100,63	101,27	98,55	99,28	99,64	100,36	100,72	101,45	96,63	98,32	99,16	100,84	101,68	103,37	94,26	97,13	98,56	101,44	102,87	105,74
-1.30	91,32	93,66	94,83	97,17	98,34	100,68	93,65	100,33	103,68	110,36	113,71	120,39	97,48	99,18	100,03	101,73	102,58	104,28	98,3	99,15	99,58	100,42	100,85	101,7
-2.00													27,24	29,43	30,53	32,71	33,81	36	37,94	44,33	47,53	53,93	57,13	63,52
-2.30	90,22	92,38	93,46	95,62	96,7	98,86	93,65	100,33	103,68	110,36	113,71	120,39	28,71	30,29	31,07	32,65	33,43	35,01	15,49	19,9	22,11	26,51	28,72	33,13
-2.60	83,67	86,19	87,45	89,97	91,23	93,75	68,14	72,71	75	79,58	81,87	86,44	26,64	28,2	28,98	30,54	31,32	32,88	24,1	27,86	29,73	33,49	35,36	39,12
-3.00	72,8	76,03	77,65	80,87	82,49	85,72	46,98	48,89	49,84	51,76	52,71	54,62												
-3.30	24,75	24,86	24,91	25,03	25,08	25,19	41,14	47,22	50,27	56,35	59,4	65,48	30,14	31,03	31,48	32,38	32,83	33,72	19,13	20,31	20,9	22,08	22,67	23,85
-4.30	20,88	21,21	21,37	21,69	21,85	22,18	8,45	8,76	8,92	9,24	9,4	9,71												

b
