# Supplementary material for: Cytotoxicity in vitro assay in 3D vs. 2D L929 cell cultures – comparative analysis of the response to the latex extracts
Source: PLoS One. 2026 Apr 28;21(4):e0347488. doi: 10.1371/journal.pone.0347488 (PMC13123997; doi:10.1371/journal.pone.0347488)
Supplement: S2 Fig — (PDF) [file pone.0347488.s002.pdf]

**NCTC clone 929 < 10 p.**

| | Norm. Mean FC | Norm. SEM |
|---|---|---|
| BAX | | |
| | 1 | 0,23 |
| | 0,98 | 0,24 |
| | 3,20 | 0,69 |
| | 6,15 | 1,71 |
| BCL-2 | | |
| | 1 | 0,19 |
| | 1,20 | 0,29 |
| | 1,75 | 0,48 |
| | 0,67 | 0,16 |
| Jkamp | | |
| | 1 | 0,15 |
| | 1,58 | 0,23 |
| | 6,30 | 2,69 |
| | 3,01 | 0,60 |
| PIDD1 | | |
| | 1 | 0,21 |
| | 1,19 | 0,27 |
| | 0,48 | 0,11 |
| | 1,71 | 0,28 |
| CYP3a44 | | |
| | 1 | 0,26 |
| | 1,12 | 0,39 |
| | 1,20 | 0,20 |
| | 17,17 | 6,37 |

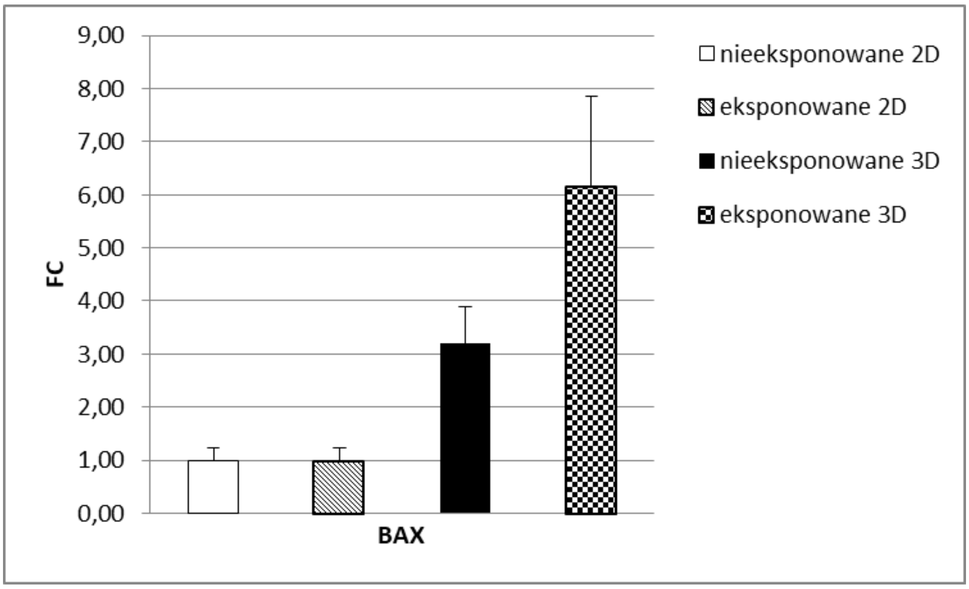

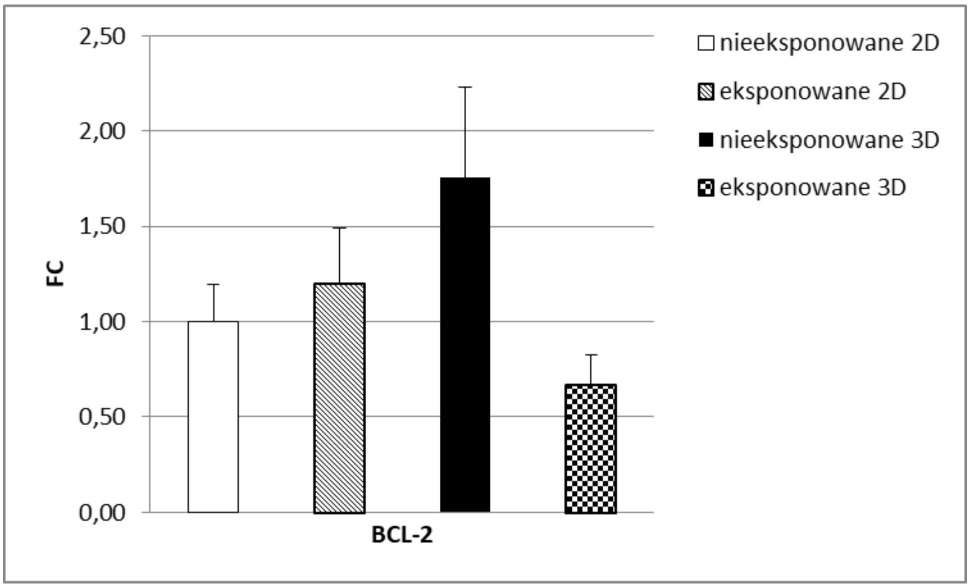
2,50
2,00
1,50
1,00
0,50
0,00
FC
BCL-2
nieeksponowane 2D
eksponowane 2D
nieeksponowane 3D
eksponowane 3D

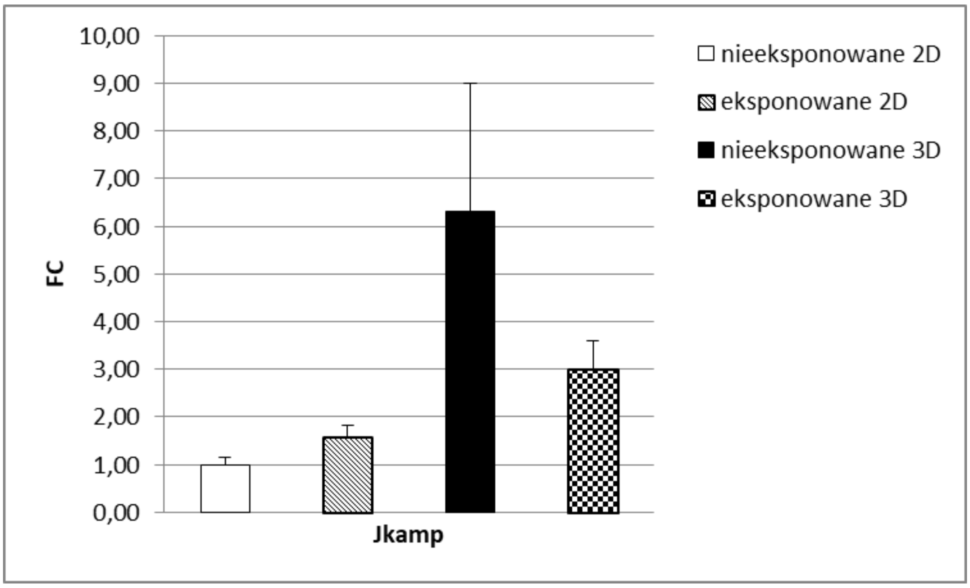
10,00
9,00
8,00
7,00
6,00
5,00
4,00
3,00
2,00
1,00
0,00
FC
Jkamp
nieeksponowane 2D
eksponowane 2D
nieeksponowane 3D
eksponowane 3D

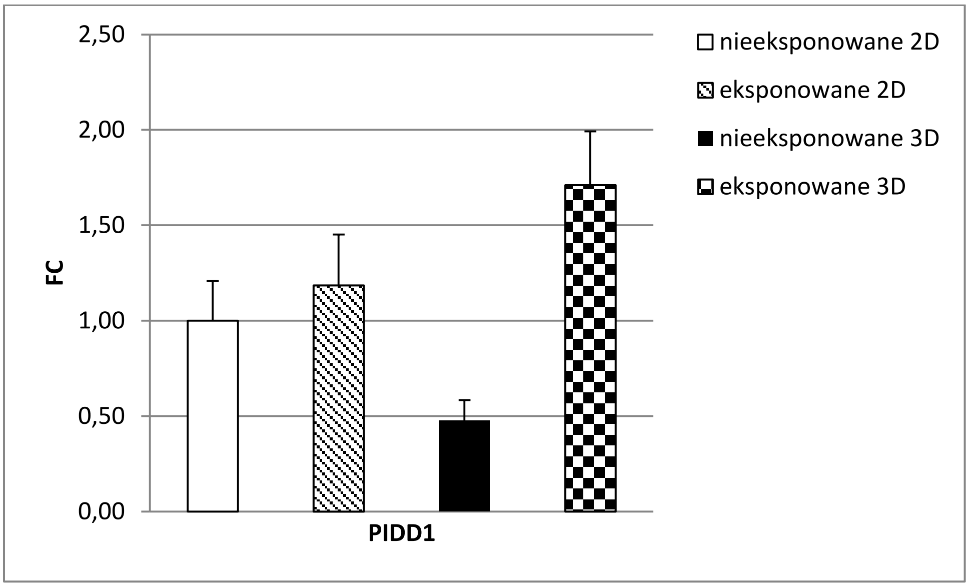


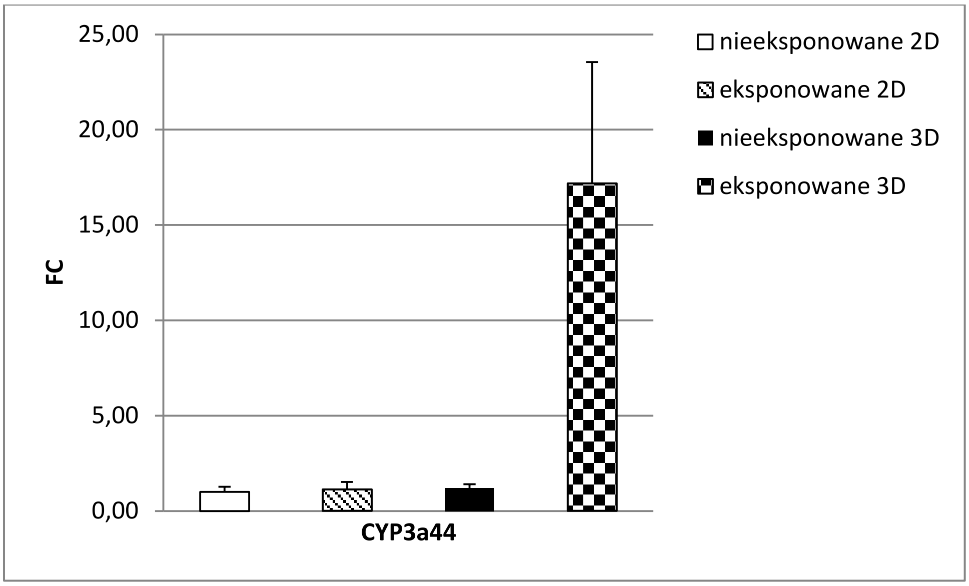


**S2_Figure a**

**NCTC clone 929 > 20 p.**

| | **norm. ŚR.** | **norm SEM** |
|---|---|---|
| | 1 | 0,25 |
| **BAX** | 1,33 | 0,13 |
| | 401,25 | 0,71 |
| | 4,10 | 0,39 |
| | | |
| | 1 | 0,19 |
| **BCL-2** | 1,20 | 0,29 |
| | 1,75 | 0,48 |
| | 0,67 | 0,16 |

| | | |
|---|---|---|
| | 1 | 0,15 |
| **Jkamp** | 1,58 | 0,23 |
| | 6,30 | 2,69 |
| | 3,01 | 0,60 |
| | | |
| | 1 | 0,21 |
| **PIDD1** | 1,19 | 0,27 |
| | 0,48 | 0,11 |
| | 1,71 | 0,28 |
| | | |
| | 1 | 0,26 |
| **CYP3a44** | 1,12 | 0,39 |
| | 1,20 | 0,20 |
| | 17,17 | 6,37 |

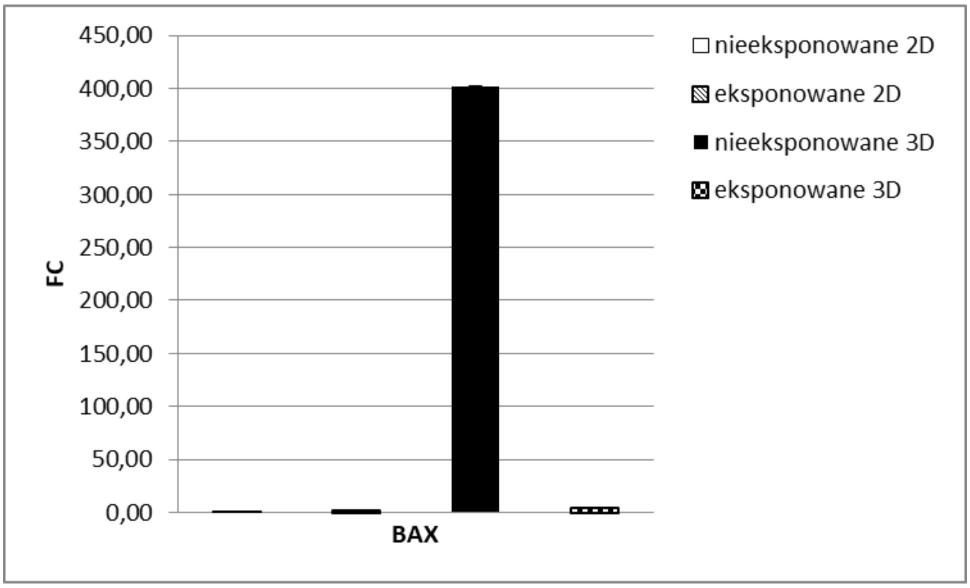

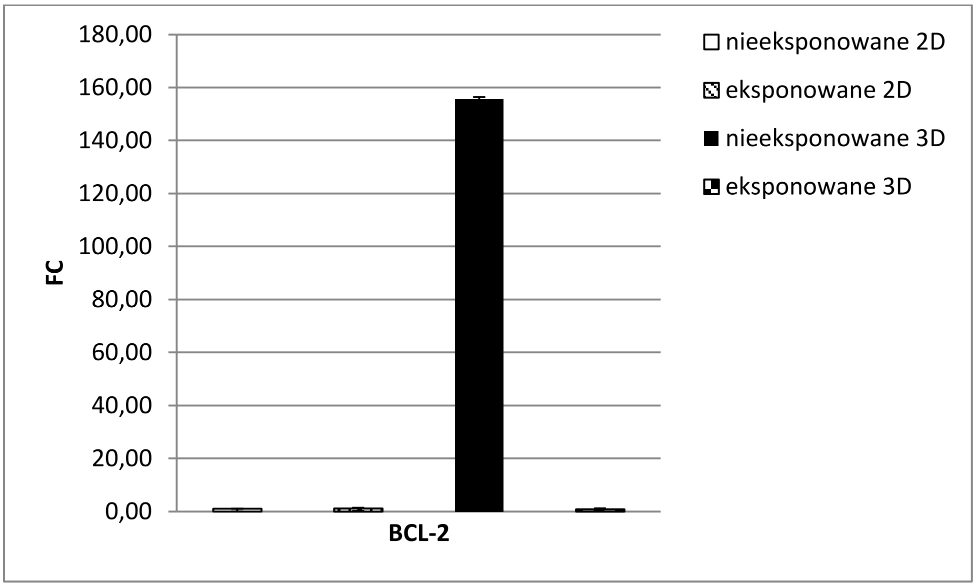
180,00
160,00
140,00
120,00
100,00
80,00
60,00
40,00
20,00
0,00
FC
BCL-2
nieeksponowane 2D
eksponowane 2D
nieeksponowane 3D
eksponowane 3D

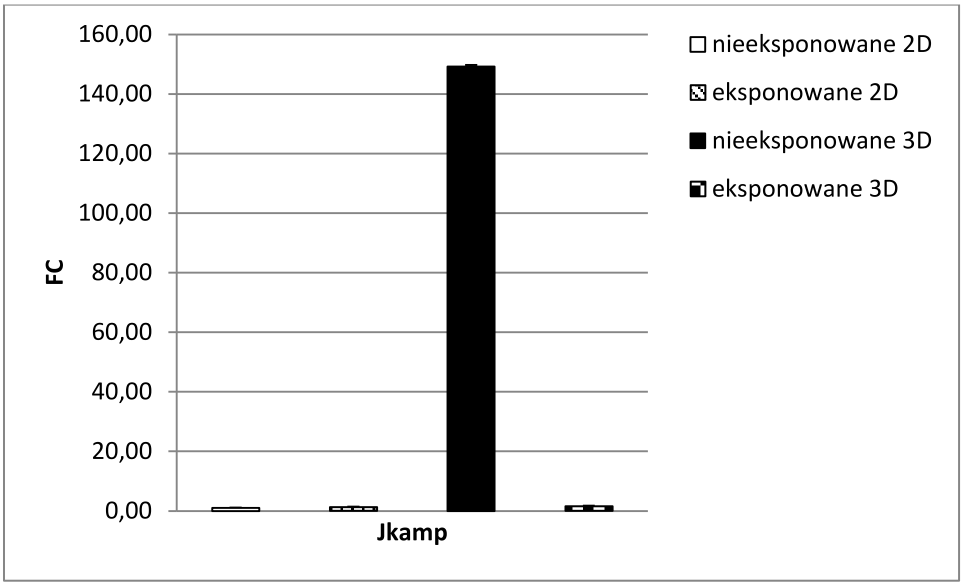
160,00
140,00
120,00
100,00
80,00
60,00
40,00
20,00
0,00
FC
Jkamp
nieeksponowane 2D
eksponowane 2D
nieeksponowane 3D
eksponowane 3D

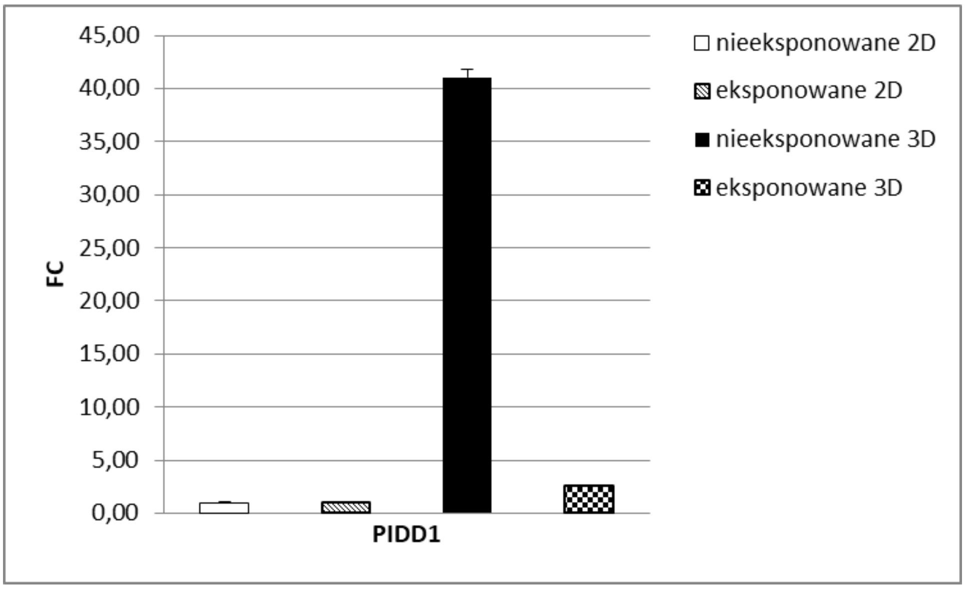
45,00
40,00
35,00
30,00
25,00
20,00
15,00
10,00
5,00
0,00
FC
PIDD1
nieeksponowane 2D
eksponowane 2D
nieeksponowane 3D
eksponowane 3D

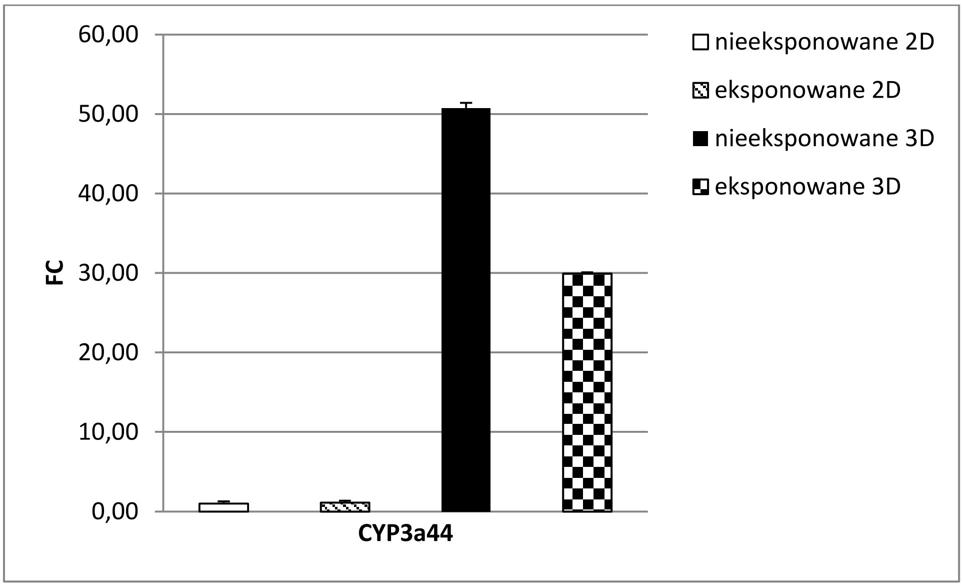


**b**

**S2 Figure**. Normalized mean FC and SEM values, developed in the Quiagen program, form the basis for generating graphs. (**a**) NCTC klon 929 < 10 p. (**b**) NCTC klon 929 > 20 p.
