# Supplementary material for: Cytotoxicity in vitro assay in 3D vs. 2D L929 cell cultures – comparative analysis of the response to the latex extracts
Source: PLoS One. 2026 Apr 28;21(4):e0347488. doi: 10.1371/journal.pone.0347488 (PMC13123997; doi:10.1371/journal.pone.0347488)
Supplement: S3 File — (PDF) [file pone.0347488.s003.pdf]

S3 Supporting information. FC calculation

Raw data from the Stratagene Mx3005P instrument, in the form of Excel files with Ct values, were analyzed on the QIAGEN website: https://dataanalysis2.qiagen.com/pcr.

An account was created for this purpose.

The following manual was used: "RT2 Profiler PCR Arrays & Assays Data Analysis Handbook."

To load the raw data onto the QIAGEN platform, Teplates were prepared using the Excel files with Ct values.
